# Hyperuricemia Predicts Adverse Outcomes After Myocardial Infarction With Non-obstructive Coronary Arteries

**DOI:** 10.3389/fmed.2021.716840

**Published:** 2021-09-09

**Authors:** Abdul-Quddus Mohammed, Fuad A. Abdu, Lu Liu, Wen Zhang, Guoqing Yin, Yawei Xu, Wenliang Che

**Affiliations:** ^1^Department of Cardiology, Shanghai Tenth People's Hospital, Tongji University School of Medicine, Shanghai, China; ^2^Department of Cardiology, Shanghai Tenth People's Hospital Chongming Branch, Shanghai, China

**Keywords:** myocardial infarction, MINOCA, serum uric acid, hyperuricemia, outcome

## Abstract

**Background:** Serum uric acid (SUA) is a well-known predictor of adverse outcomes in patients with various clinical conditions. However, the impact of SUA on patients with myocardial infarction with non-obstructive coronary arteries (MINOCA) remains unclear. Here, we aimed at investigating the potential association between hyperuricemia and the adverse outcomes in MINOCA patients.

**Methods:** Overall, 249 MINOCA patients were enrolled in the present study. Clinical characteristics and laboratory data, were measured in all patients. Based on SUA levels, patients were classified into two groups; the hyperuricemia group [SUA level > 6 mg/dL (360 μmol/L) in women and > 7 mg/dL (420 μmol/L) in men], and the normuricemia group. The primary endpoint of our study was major adverse cardiac events (MACE), defined as cardiovascular death, stroke, heart failure, non-fatal MI, and angina rehospitalization.

**Results:** Seventy-two patients were in hyperuricemia group and 177 in normuricemia group. Fifty-two MACE events were recorded after 30 months of follow-up period. The incidence of MACE was higher in hyperuricemia group compared with normuricemia group (31.9 vs. 16.3%, *P* = 0.006). Kaplan-Meier survival curves illustrated a significantly increased risk of MACE in hyperuricemia group (log-rank *P* = 0.006). The multivariable logistic analysis demonstrated that hyperuricemia was independently associated with a high risk of MACE after 30 months of follow-up (OR, 2.234; 95% CI, 1.054–4.737, *P* = 0.036).

**Conclusion:** Hyperuricemia is associated with adverse outcomes and appears to be an independent predictor of MACE in MINOCA patients. This finding suggests that the SUA levels may serve as a surrogate biomarker related to risk prediction and adverse outcomes of MINOCA patients.

## Introduction

Despite great advances in our undertanding of cardiovascular diseases, myocardial infarction with non-obstructive coronary arteries (MINOCA) remains an intriguing clinical entity which has gained increased attention over the recent years, comprises 5–15% of overall acute myocardial infarction (AMI) cases (1, 2). MINOCA is not a benign entity with multiple underlying pathological mechanisms, and it is associated with a higher risk of a major adverse cardiac event (MACE) leading to early rehospitalization, with an in-hospital mortality and 5 years mortality rate of 4.57 and 10.9%, respectively (3–6). Therefore, identifying new biomarkers for adverse outcome associated to patients with MINOCA would be of potential interest, as they represent a heterogenous group and could be monitored with caution and treated with an appropriate preventive strategy.

Serum uric acid (SUA) represents the end-product of purine catabolism (7). Elevated SUA is now considered to have a potential relationship with multiple clinical conditions (8) particularly, cardiovascular diseases (9, 10). Emerging research also correlates hyperuricemia with enhanced intracellular oxidative stress, inflammation and vasoconstriction, as well as endothelial dysfunction (7, 11, 12) as a result, atherosclerosis and cardiovascular disease progression (13). In addition, elevated SUA is implicated as a marker of poor outcomes in patients with arterial hypertension (14), metabolic syndrome (15), coronary artery disease (CAD) (16), cerebrovascular disease (17), myocardial infarction (MI) (18), heart failure (HF) (19), renal disease (10, 20) and in general population (21, 22). However, the role of hyperuricemia in patients with MINOCA, as well as whether hyperuricemia is associated with adverse outcomes has not been studied.

Therefore, the present study sought to explore the association between hyperuricemia and adverse outcomes in MINOCA patients.

## Methods

### Study Population

This was an observational study which was carried out at the department of cardiology, Shanghai Tenth People's Hospital, between 2014 and 2018. We enrolled 249 consecutive patients who underwent coronary angiography (CAG) and were diagnosed with MINOCA.

MINOCA was identified as patients who had evidence of AMI with non-obstructive coronary artery disease (defined as no stenosis; or stenosis <50%) as recommended by the current position statement from the European Society of Cardiology (ESC) working group and the fourth Universal Definition of MI (1, 2). We excluded patients who had a prior history of MI or coronary intervention, severe liver disease, gout, and a malignant tumor; patients with typical myocarditis or Takotsobu syndrome; patients who were unable to obtain SUA determination; and patients who do not have SUA baseline results.

After admission, demographic and baseline clinical data [such as age, sex, smoking history, blood pressure, heart rate, diabetes, hypertension, BMI, hyperlipidemia, chronic kidney disease (CKD) and atrial fibrillation], and diuretic were collected. In addition, all patients following their entry underwent an electrocardiogram, echocardiography, and CAG procedure.

Fasting blood was obtained within 24 h of hospitalization. The serum uric acid (SUA) levels, serum cardiac biomarkers [such as troponin-T (Tn-T), myoglobin, N-terminal-pro-brain natriuretic peptide (NTproBNP), and creatine kinase-isoenzyme MB (CK-MB)], lipid panel measures [such as total cholesterol (TC), triglyceride (TG), high-density lipoprotein cholesterol (HDL-C), low-density lipoprotein cholesterol (LDL-C)] and estimated glomerular filtration rate (eGFR) were measured in all patients.

SUA levels were measured by Olympus AU4500 automatic chemistry analyzer (Olympus Corporation, Tokyo, Japan). GFR was estimated using a Modification of Diet in Renal Disease (MDRD) method, and CKD is defined as eGFR <60 ml/min/1.73 m^2^ (23). Comorbidities such as hypertension, dyslipidemia and diabetes were described as either previously known or if patients are on specific therapy. NTproBNP levels were measured by the Eleusis electro-chemiluminescent immunoassay (Roche Diagnostics Ltd. Rotkreuz, Switzerland).

Left ventriculography and echocardiography have been used to evaluate wall motion, and intravascular ultrasound or optical coherence tomography were utilized to assess atherosclerotic plaque disruption or plaque erosion, coronary angiography derived flow fractional reserve was performed in selected patients to evaluate the functional significance of coronary artery lesions in the present study to investigate the ultimate causes of MINOCA patients.

Based on SUA levels, patients were divided into two groups: the normuricemia group and the hyperuricemia group. Hyperuricemia was defined as SUA levels > 6 mg/dL (360 μmol/L) in women and > 7 mg/dL (420 μmol/L) in men, as previously published (24, 25).

The study was approved by the institutional ethics committee (Shanghai Tenth People's Hospital, Tongji University, Shanghai, China) and was complied in accordance with the Helsinki Declaration. Each enrolled participant in this study signed an informed consent form.

### Follow-Up

Follow-up was carried out for 30 months after discharge through outpatient visits, telephone calls, reviewing electronic medical records, and clinical notes by two experienced cardiologists to obtain the patient's clinical status and outcome events or the first reported outcome case at the Shanghai Tenth People's Hospital.

The study primary endpoint was MACE, defined as cardiovascular death, stroke, heart failure, non-fatal MI, and angina rehospitalization. Cardiovascular death was described as death due to the cardiac origin, including acute coronary syndrome (ACS), severe arrhythmias, refractory congestive heart failure, or sudden death with no obvious cause. Non-fatal MI was defined as characteristic signs and symptoms of myocardial ischemia in the presence of new ECG changes or increased levels of cardiac biomarkers of myocardial damage (2). Stroke was defined as evidence of ischemic infarct in any cerebral artery caused by either thrombotic or an embolic occlusion (26). The definition of heart failure was in accordance with the previous HF guidelines (27). Angina rehospitalization was defined as any rehospitalization or readmission to emergency department due to recurrent ischemic discomfort with objective evidence recorded by the physician.

### Statistical Analysis

Data were processed using the Statistical Package for Social Sciences (SPSS) v.22. The categorical variables are expressed as percentages (%), and numbers were expressed as the mean ± SE. To compare the categorical variables, the chi-square test and the Fisher's exact tests were performed. An independent sample *t*-test was performed when numerical variables were compared between the groups. Multivariable logistic regression analysis was performed to explore the adjusted odds ratio (OR) for MACE events to determine the predictors of clinical endpoints. Traditional cardiovascular risk factors (e.g., age, sex, hypertension, diabetes, BMI, atrial fibrillation, hyperlipidemia, CKD, and LVEF), biochemical parameters [serum levels of uric acid (categorical variables), Tn-T, myoglobin, NTproBNP, TG, TC, LDL-C and HDL-C], and diuretic use were considered as covariates in the univariate models. Univariate predictors (when *P* <0.10) were variables in covariates for multivariable models. Kaplan-Meier analysis was used to assess MACE-free survival rates, and the log-rank test was performed to identify differences between groups. Furthermore, the association between SUA levels and the risk of MACE was also evaluated using restricted cubic spline Cox regression. To assess potential relationship between hyperuricemia and MACE, subgroup analyses were performed, and interactions between hyperuricemia and each subgroup including age, sex, hypertension, diabetes, dyslipidemia, smoking, atrial fibrillation, LVEF, and eGFR levels were evaluated by a Cox proportional hazards regression model. All statistical analyses were performed two-sided and required a statistical significance of *P*-value <0.05.

## Results

### Baseline Characteristics of the Study Population

Baseline characteristics and angiographic data of the study population stratified by the presence of hyperuricemia are summarized in Table 1. Overall, 249 patients were eligible and identified for the diagnostic criteria of MINOCA (mean age, 62.67 ± 13.58 years). Seventy-two patients (28.9%) were in the hyperuricemia group and 177 patients (71.1%) in the normuricemia group. Compared with normuricemia group, hypertension, CKD and atrial fibrillation were more frequent in hyperuricemia group, BMI and diuretic use were also higher in hyperuricemia group, whereas eGFR and LVEF values were lower. By comparison, the normuricemia group had a higher rate of females than hyperuricemia group. Coronary angiography data revealed that patients in hyperuricemia group had a higher rate of mild coronary stenosis than normuricemia group.

**Table 1:** Baseline characteristics of the study population.

	**Hyperuricemia(*n* =72)**	**Normuricemia(*n* =177)**	***P*-value**
Age (years)	64.31 ± 14.97	62.00 ± 12.96	0.226
Female, *n* (%)	27 (37.5)	95 (53.7)	0.021
Hypertension, *n* (%)	44 (61.1)	77 (43.5)	0.012
Diabetes, *n* (%)	14 (19.4)	27 (15.3)	0.419
Smoking history, *n* (%)	35 (48.6)	65 (36.7)	0.083
BMI (kg/m^2^)	25.08 ± 4.24	23.68 ± 3.61	0.012
Atrial fibrillation, *n* (%)	13 (18.1)	14 (7.9)	0.020
Chronic kidney disease	30 (41.7)	17 (9.6)	<0.001
Hyperlipidaemia, *n* (%)	16 (22.2)	23 (13.0)	0.069
eGFR, ml/min	70.38 ± 28.66	94.41 ± 26.42	<0.001
LVEF (%)	51.03 ± 13.91	56.21 ± 10.46	0.002
STEMI, *n* (%)	30 (41.7)	71 (40.1)	0.821
Systolic blood pressure (mmHg)	138.04 ± 25.29	141.69 ± 22.12	0.259
Diastolic blood pressure (mmHg)	79.83 ± 14.48	80.54 ± 12.45	0.698
Heart rate, beats per minute	84.31 ± 20.95	80.75 ± 16.55	0.157
Diuretic	35 (48.6)	35 (19.8)	<0.001
**Angiographic data**			
Normal coronary arteries (0% stenosis), *n* (%)	21 (29.2)	97 (54.8)	<0.001
Mild coronary stenosis (stenosis <50%), *n* (%)	51 (70.8)	80 (45.2)	<0.001

*BMI, body mass index; LVEF, left ventricular ejection fraction; eGFR, estimated Glomerular filtration rate; STEMI, ST-segment elevation myocardial infarction*.

Laboratory analysis of the study population is summarized in Table 2. Hyperuricemia group had higher levels of TG and myocardial biomarkers such as myoglobin and NTproBNP compared with the normuricemia group (all *P* <0.05). In contrast, other comorbidities such as age, smoking history, hyperlipidemia, diabetes, heart rate, systolic and diastolic blood pressure, and other laboratory findings appeared to be similar between the two groups (all *P* > 0.05).

**Table 2:** Laboratory findings of the study population.

	**Hyperuricemia(*n* =72)**	**Normuricemia(*n* =177)**	***P*-value**
Uric acid, μmol/L	489.17 ± 99.25	296.84 ± 72.33	<0.001
Tn-T (ng/mL)	0.48 ± 1.27	0.50 ± 1.06	0.895
CK-MB (ng/mL)	23.96 ± 67.02	17.94 ± 34.77	0.354
Myoglobin (ng/ml)	211.57 ± 411.52	113.59 ± 190.81	0.011
NTproBNP (pg/mL)	3,796.49 ± 6,378.48	1,576.58 ± 3,773.46	0.001
TC (mmol/L)	4.33 ± 1.25	4.22 ± 0.99	0.462
TG (mmol/L)	1.73 ± 1.21	1.35 ± 0.71	0.004
HDL-C (mmol/L)	1.02 ± 0.49	1.05 ± 0.49	0.657
LDL-C (mmol/L)	2.47 ± 1.06	2.47 ± 0.87	0.977

*CK-MB, creatine kinase-isoenzyme; Tn-T, troponin T; NTproBNP, N-terminal-pro-brain natriuretic peptide; TC, total cholesterol; TG, triglyceride; HDL-C, high-density lipoprotein cholesterol; LDL-C, low-density lipoprotein cholesterol*.

### Hyperuricemia and Clinical Outcomes

**Figure 1:**
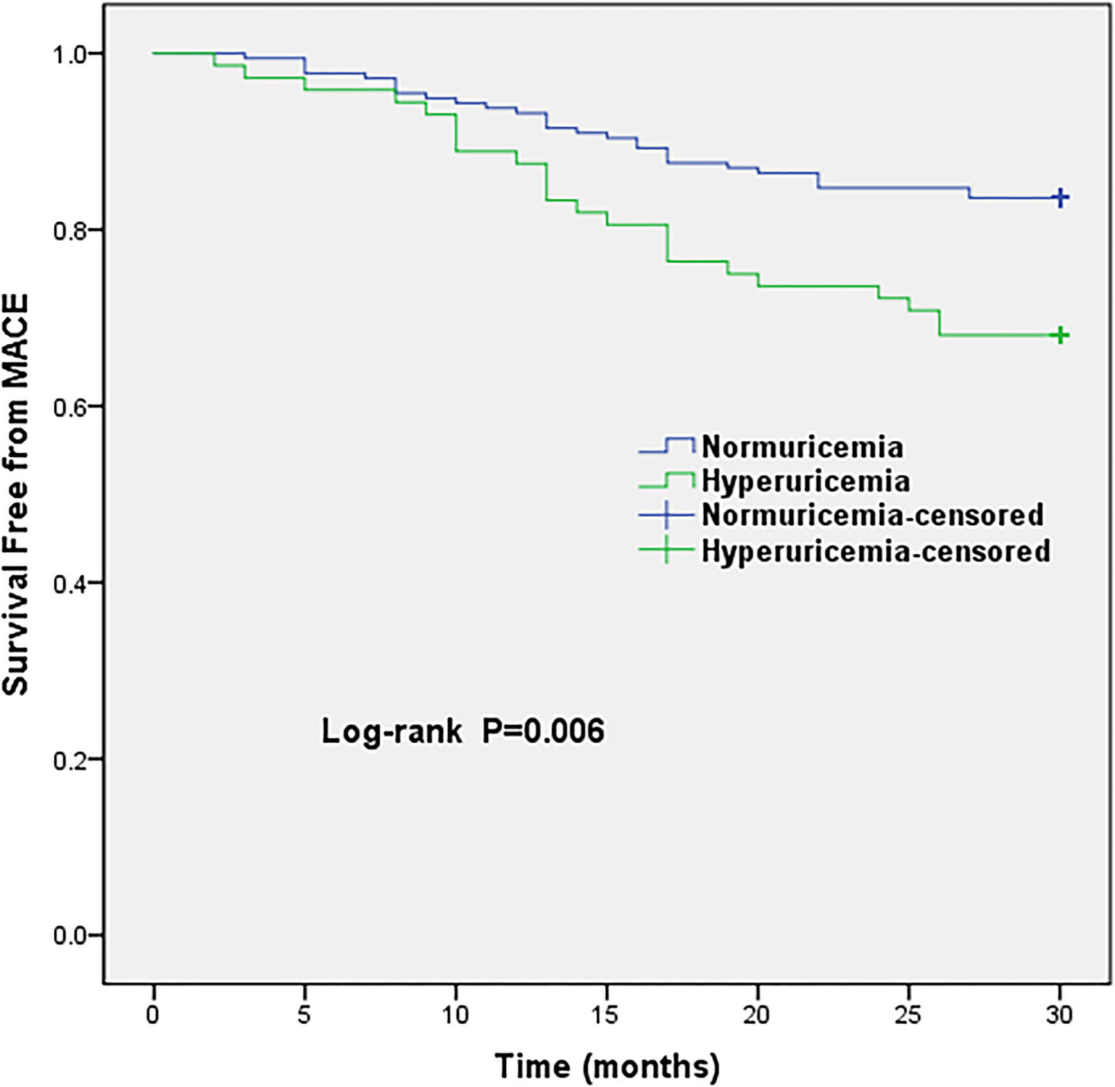
Kaplan-Meier survival curves of MACE in MINOCA patients with hyperuricemia vs. normuricemia. MACE, major adverse cardiovascular events.

The average follow-up period was 30 months. All patients were available with follow-up data, 52 MACE were recorded. Hyperuricemia group was associated with 23 MACE, whereas, 29 MACE occurred in the normuricemia group. The hyperuricemia group had a higher incidence of MACE and angina rehospitalization compared with normuricemia group (31.9 vs. 16.3% and 19.4 vs. 9.0%, respectively; all *P* <0.05) (Table 3). Increased risk of MACE in hyperuricemia group is shown in Kaplan-Meier survival curves, which demonstrated that MINOCA patients within the hyperuricemia group were clearly separated from the normuricemia group, and the significant statistical difference is noted (log-rank *P* = 0.006) (Figure 1). Additionally, the restricted cubic splines revealed a linear relationship between SUA levels and MACE, indicating that the risk of MACE occurrence increased gradually with continuous increase in SUA levels (P for non-linearity = 0.806) (Supplementary Figure 1).

**Table 3:** Patients outcomes.

	**Hyperuricemia(n =72)**	**Normuricemia(n =177)**	***P-*value**
MACE	23 (31.9)	29 (16.3)	0.006
Cardiovascular death	5 (6.9)	9 (5.1)	0.554
Nonfatal MI	1 (1.4)	1 (0.5)	0.496
Heart failure	1 (1.4)	2 (1.1)	1.000
Stroke	2 (2.8)	1 (0.5)	0.201
Angina rehospitalization	14 (19.4)	16 (9.0)	0.022

*MACE, major adverse cardiovascular events; MI, myocardial infarction*.

The predictors of MACE from the univariate and multivariable analysis are shown in Table 4. Univariate logistic analysis showed that hyperuricemia was independent predictor of MACE in MINOCA patients (OR, 2.395; 95% CI, 1.269–4.523, *P* = 0.007). Furthermore, after adjusting for potential confounders, multivariable logistic analysis persistently demonstrated that hyperuricemia was associated with an increased risk of MACE over the follow-up period of 30 months (OR, 2.234; 95% CI, 1.054–4.737, *P* = 0.036).

**Table 4:** Univariate and multivariable analysis of predictors of MACE.

**Variables**	**Univariate analysis**		**Multivariable analysis**	
	**OR (95% CI)**	***P*-value**	**OR (95% CI)**	***P*-value**
Hyperuricemia	2.395 (1.269–4.523)	0.007	2.234 (1.054–4.737)	0.036
Hypertension	2.151 (1.145–4.041)	0.017		
Diabetes	2.012 (0.956–4.235)	0.066		
Age	1.038 (1.013–1.064)	0.003	1.029 (1.000–1.058)	0.048
LVEF	0.963 (0.940–0.987)	0.002	0.966 (0.938–0.995)	0.022
CKD	2.078 (1.022–4.224)	0.043		
Diuretic use	1.838 (0.965–3.499)	0.064		

*LVEF, left ventricular ejection fraction; CKD, chronic kidney disease; CI, confidence interval; OR, odds ratio*.

The associations between hyperuricemia and MACE stratified by sex, age, hypertension, diabetes, dyslipidemia, smoking, atrial fibrillation, LVEF, and eGFR levels are shown in Table 5, which demonstrated that there were no interactions between hyperuricemia and clinically related variables except for an interaction with hypertension (*P* = 0.048); HR of 1.026 (95% CI, 0.379–2.773, *P* = 0.960) with hypertension, and HR of 4.314 (95% CI, 1.564–11.906, *P* = 0.005) without hypertension.

**Table 5:** Subgroup analysis of the association between hyperuricemia and adverse outcomes.

**Factors**	**Subgroup**	**HR (95% CI)**	**Interaction *P*-value**
Age	<65 years	2.396 (0.865–6.582)	0.677
	≥65 years	1.769 (0.673–4.648)	
Sex	Male	2.963 (1.055–8.327)	0.412
	Female	1.624 (0.571–4.609)	
Hypertension	Yes	1.026 (0.379–2.773)	0.048
	No	4.314 (1.564–11.906)	
Diabetes	Yes	1.646 (0.275–9.863)	0.784
	No	2.155 (1.008–4.603)	
Smoking	Yes	3.238 (0.913–11.481)	0.408
	No	1.741 (0.731–4.152)	
Atrial fibrillation	Yes	0.431 (0.045–4.145)	0.137
	No	2.538 (1.208–5.335)	
Hyperlipidaemia	Yes	0.503 (0.052–4.839)	0.175
	No	2.596 (1.230–5.435)	
LVEF	<50	2.147 (0.744–6.191)	0.636
	≥50	1.519 (0.570–4.049)	
eGFR	<60	2.784 (0.591–13.119)	0.488
	≥60	1.466 (0.574–3.747)	

*BMI, body mass index; LVEF, left ventricular ejection fraction; eGFR, estimated Glomerular filtration rate; HR, hazard ratio; CI, confidence interval*.

## Discussion

The present study evaluated whether hyperuricemia was associated with adverse outcomes in patients with MINOCA. Our study found that hyperuricemia is associated with adverse outcomes and appears to be an independent predictor of MACE in MINOCA patients, which suggests that quantification of SUA is an appropriate measure of predicting adverse outcomes following MINOCA.

MINOCA is a distinct type of MI that has received a considerable amount of interest as it was recently introduced in the ESC and the fourth Universal Definition of MI (2018) guidelines (1, 2). MINOCA is described as a non-benign entity with multiple underlying pathological etiologies leading to management inconsistency. The in-hospital mortality rate was 4.6% in a Japanese study of 13022 MINOCA patients (5). A recent multi-center cohort study in 16849 MINOCA patients reported an 18.7% rate of MACE events over 12 months, one out of every five MINOCA patients suffered a major adverse event (28). Therefore, additional risk stratification to further refine new clinical predictive factors is essential to identify MINOCA patients who are at increased risk of new MACE events.

During the past decade, there has been an increasing awareness that hyperuricemia is associated with potential cardiovascular diseases (9). Noteworthy, the relation to its prevalence varied among the studies due to diverse definitions of hyperuricemia. In patients with ACS, the prevalence of hyperuricemia was found to be 34.4 and 29.3% (29, 30). Consistent with previous studies, the present study found a 28.9% prevalence rate of hyperuricemia in MINOCA patients.

Data among short and long-term prognosis in ACS patients demonstrated an independent association between hyperuricemia and adverse outcomes, and indicated that hyperuricemia can predict in-hospital adverse outcomes, as well as cardiovascular and all-cause mortality (29–33). Consistently, the potential role of hyperuricemia in predicting adverse prognosis is also supported by findings from AMI studies (34, 35). SUA was a strong predictor for in-hospital mortality among ST elevation myocardial infarction (STEMI) patients submitted to primary PCI (18). In a long-term follow-up of 8.4 years, the Rotterdam study found major adverse events were independently associated with hyperuricemia (36). Hyperuricemia also independently predicted the risk of mortality and MACE in patients who underwent cardiac revascularization and cardiac valve surgery (37). Two recent MINOCA studies reported that renal impairment was associated with mortality in MINOCA patients (38, 39), the latter study also found that every third patient with MINOCA is diagnosed with impaired kidney function, which implies that the uric acid may play a significant role related to the extent of impaired cardiovascular and/or renal hemodynamics in patients with MINOCA. To our knowledge, no prior study has evaluated the association between hyperuricemia and MINOCA and its impact on clinical outcomes. Our study provides new insights into the relationship between hyperuricemia and the adverse outcomes of MINOCA patients. In the present study, we observed that MINOCA patients in hyperuricemia group had a higher incidence of MACE and angina rehospitalization compared with normuricemia group. Accordingly, the Kaplan-Meier survival curves illustrated that the hyperuricemia group had a significantly higher risk of total MACE. Moreover, multivariable logistic regression showed that MINOCA patients in the hyperuricemia group demonstrated a significant association with adverse outcome, which implies that SUA levels may serve as an important index of poor prognosis in high-risk MINOCA patients.

In addition, hyperuricemia group had higher prevalence of hypertension, hypertriglyceridemia, BMI, and AF which has been correlated with elevated SUA level by earlier studies (15, 40). A potential relationship between hyperuricemia and CAD incidence and adverse events has been reported earlier (41), moreover, SUA was significantly associated with cardiovascular and all-cause mortality and revealed a “J-shaped” pattern in patients with CAD (42). In early-onset CAD, hyperuricemia was linked to the severity of CAD among patients with unstable angina and MI (43). Compared with low uric acid levels, hyperuricemia was independently associated with a 6-fold higher incidence of HF in the general population (19), as well as abnormal LVEF (44). Thus, hyperuricemia exerts a potential role in developing atherosclerotic diseases and their risk factors leading to adverse cardiovascular events (7, 12, 13). Our findings are consistent with these studies indicating that MINOCA patients in hyperuricemia group are associated with low LVEF levels and higher NT-proBNP and myoglobin levels compared to the normuricemia group; moreover, coronary arteries with mild stenosis was more frequent in hyperuricemia group, suggesting that hyperuricemia is correlated with the degree of myocardial injury and severity of MINOCA patients. Nevertheless, concomitant use of diuretics in patients with clinical conditions such as heart failure and/or renal impairment may alter serum uric acid levels, consequently affecting clinical outcomes. In the present study, even after adjustment for diuretics intake, multivariate logistic regression analysis still stated that hyperuricemia was associated with increased risk of MACE in MINOCA patients, large prospective studies are required to confirm this finding.

Taken together, the findings in the present study highlight the potential clinical significance of SUA on cardiovascular risk in MINOCA patients, if added in the risk stratification of MINOCA patients at an early stage, may prevent MACE and improve treatment options and overall outcomes in high-risk individuals. In addition, SUA levels are shown to be clinically important estimates of predicting poor prognosis. Therefore, SUA may serve as a surrogate biomarker to improve clinical risk classification in patients following MINOCA. Furthermore, hyperuricemia is a potentially treatable factor, uric acid is collected routinely in clinical practice and is inexpensive to obtain. However, it remains unclear whether modulating SUA and improving cardiovascular outcomes has clinical benefits.

Few potential limitations were related to this study. First, this was a single-centered, small retrospective-observational study with a short follow-up period. Second, data regarding SUA was available only at the admission; as a result, we are unable to provide any details on the impact of SUA level change over the follow-up period. Third, treatment with Xanthine Oxidase (XO) inhibitors which may affect the patients clinical outcomes were lacking; nevertheless, additional research warrants the effects of XO inhibitors in lowering SUA and improving cardiovascular outcomes as previous reports implicated mixed results; therefore, lowering SUA to improving cardiovascular outcomes in MINOCA patients needs further verification. Furhermore, although hyperuricemia was associated with adverse clinical outcomes after adjusting for multiple potential confounding factors, the unmeasured confounding exposure effects still cannot be entirely omitted. In addition, our results provided from one center cannot be extrapolated to different populations for general medical practice. Further research with a larger cohort and multi-center prospective studies may warrant establishing the role of uric acid and cardiovascular risk among MINOCA patients who represent a diverse phenotype and further elucidate the precise mechanisms.

## Conclusion

This is the first study investigating the impact of SUA on MACE in MINOCA patients. Our study demonstrated that hyperuricemia is associated with adverse outcomes and appears to be an independent predictor of MACE in MINOCA patients. This finding suggests that the SUA levels may serve as a surrogate biomarker related to risk prediction and adverse outcomes of MINOCA patients. Whether modulating UA in MINOCA may benefit clinically requires further elucidation.

## Data Availability Statement

The raw data supporting the conclusions of this article will be made available by the authors, without undue reservation.

## Ethics Statement

The studies involving human participants were reviewed and approved by Shanghai Tenth People's Hospital, Tongji University, Shanghai, China. The patients/participants provided their written informed consent to participate in this study.

## Author Contributions

A-QM, FA, and WC contributed to the conception and designed of the research. Data acquisition, analysis and interpretation were performed by LL, GY, A-QM, and FA collected the data. WZ was involved in data cleaning, follow-up, and verification. A-QM, FA, YX, and WC drafted and revised the manuscript critically for important intellectual content. WC and FA approved the final version of the manuscript. The final version to be published was approved by all authors.

## Funding

This work was supported by Chinese National Natural Science Foundation (82170521), Shanghai Natural Science Foundation of China (21ZR1449500), the Fundamental Research Funds for Central Universities (NO.22120190211), Foundation of Chongming (CKY2021-21 and CKY2020-29), and Clinical Research Plan of SHDC (SHDC2020CR4065).

## Conflict of Interest

The authors declare that the research was conducted in the absence of any commercial or financial relationships that could be construed as a potential conflict of interest.

## Publisher's Note

All claims expressed in this article are solely those of the authors and do not necessarily represent those of their affiliated organizations, or those of the publisher, the editors and the reviewers. Any product that may be evaluated in this article, or claim that may be made by its manufacturer, is not guaranteed or endorsed by the publisher.

## Abbreviations

SUA: Serum uric acid
AMI: acute myocardial infarction
MINOCA: myocardial infarction with non-obstructive coronary arteries
ACS: acute coronary syndrome
MACE: major adverse cardiac events
eGFR: estimated Glomerular filtration rate
CAG: coronary angiography
CK-MB, creatine kinase-isoenzyme, Tn-T: troponin T
NTproBNP: N-terminal-pro-brain natriuretic peptide
BMI: body mass index
LVEF: left ventricular ejection fraction
CI: confidence interval
OR: odds ratio
TG: triglyceride
TC: total cholesterol
HDL-C: high-density lipoprotein cholesterol
LDL-C: low-density lipoprotein cholesterol.
