# Supplementary figures and images for: Hyperuricemia Predicts Adverse Outcomes After Myocardial Infarction With Non-obstructive Coronary Arteries
Source: Front Med (Lausanne). 2021 Sep 9;8:716840. doi: 10.3389/fmed.2021.716840 (PMC8458886; doi:10.3389/fmed.2021.716840)

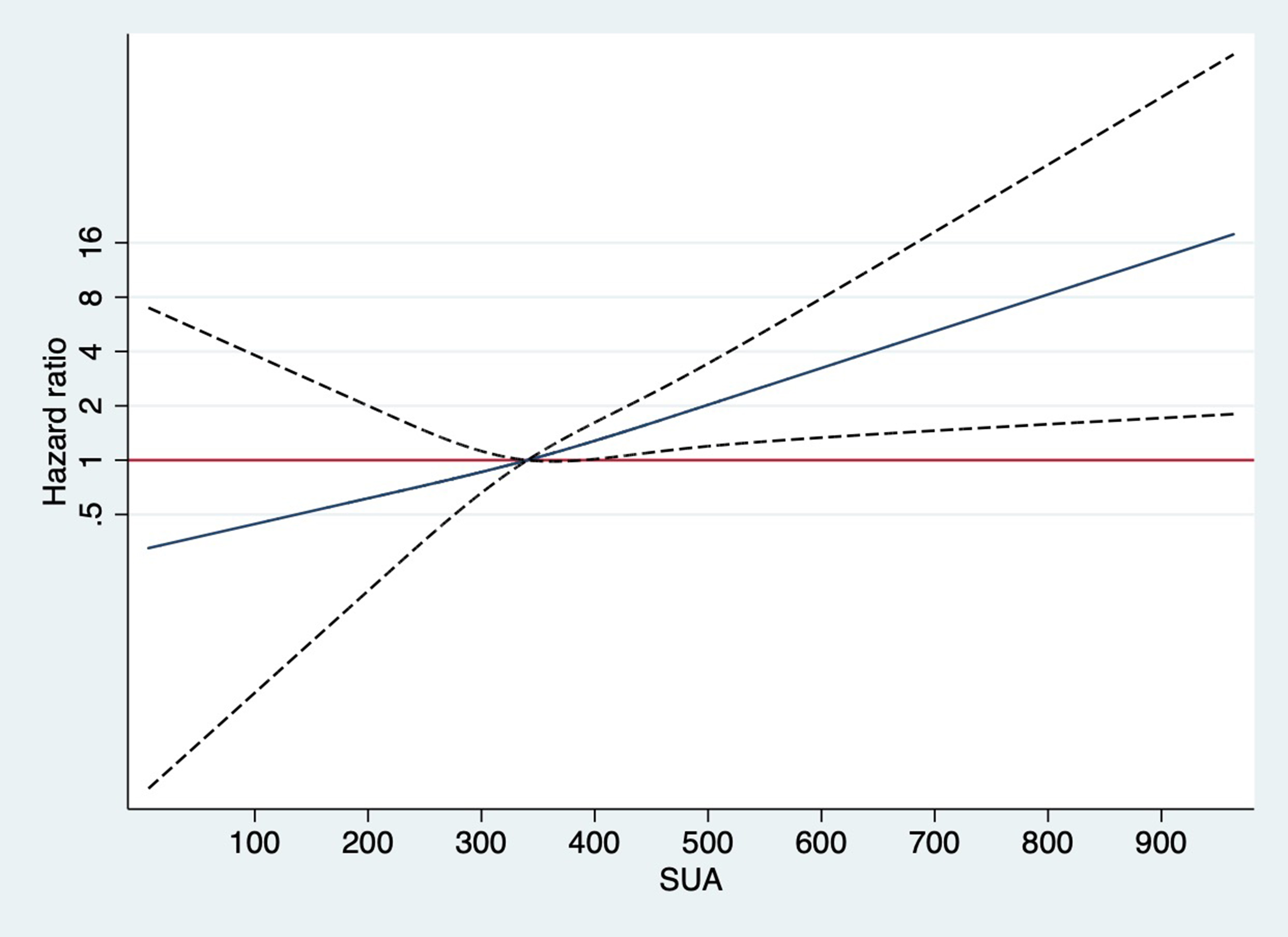

Supplement: Supplementary file 1 [file Image_1.JPG]
